# Polyamine supplementation reduces DNA damage in adipose stem cells cultured in 3-D

**DOI:** 10.1038/s41598-019-50543-z

**Published:** 2019-10-03

**Authors:** Manuela Minguzzi, Serena Guidotti, Daniela Platano, Stefania D’Adamo, Silvia Cetrullo, Elisa Assirelli, Spartaco Santi, Erminia Mariani, Giovanni Trisolino, Giuseppe Filardo, Flavio Flamigni, Rosa Maria Borzì

**Affiliations:** 1 Dipartimento di Scienze Mediche e Chirurgiche, Università di Bologna, Bologna, Italy; 2 Laboratorio di Immunoreumatologia e Rigenerazione Tissutale, IRCCS Istituto Ortopedico Rizzoli, Bologna, Italy; 3 Dipartimento di Scienze Biomediche e Neuromotorie, Università di Bologna, Bologna, Italy; 4 Institute of Molecular Genetics, National Research Council (CNR) c/o IRCCS Istituto Ortopedico Rizzoli, Bologna, Italy; 5 Struttura Complessa di Ortopedia e Traumatologia Pediatrica, IRCCS Istituto Ortopedico Rizzoli, Bologna, Italy; 6 Applied and Translational Research Center, IRCCS Istituto Ortopedico Rizzoli, Bologna, Italy

**Keywords:** Autophagy, Mesenchymal stem cells

## Abstract

According to previous research, natural polyamines exert a role in regulating cell committment and differentiation from stemness during skeletal development. In order to assess whether distinct polyamine patterns are associated with different skeletal cell types, primary cultures of stem cells, chondrocytes or osteoblasts were dedicated for HPLC analysis of intracellular polyamines. Spermine (SPM) and Spermidine (SPD) levels were higher in adipose derived stem cells (ASC) compared to mature skeletal cells, i.e. chondrocytes and osteoblasts, confirming the connection of polyamine content with stemness. To establish whether polyamines can protect ASC against oxidative DNA damage in a 3-D differentiation model, the level of γH2AX was measured by western blot, and found to correlate with age and BMI of patients. Addition of either polyamine to ASC was able to hinder DNA damage in the low micromolecular range, with marked reduction of γH2AX level at 10 µM SPM and 5 µM SPD. Molecular analysis of the mechanisms that might underlie the protective effect of polyamine supplementation evidences a possible involvement of autophagy. Altogether, these results support the idea that polyamines are able to manage both stem cell differentiation and cell oxidative damage, and therefore represent appealing tools for regenerative and cell based applications.

## Introduction

Osteoarthritis (OA), the structural and functional failure of articular cartilage, represents the leading reason of chronic disability in aged people that heavily affects patients’ quality of life and national health systems’ expenditure. OA progresses until the complete loss of hyaline cartilage^1^. To date no “disease modifying” therapy able to slow or revert disease progression is available. In the field of regenerative therapy, adipose tissue is a convenient reservoir of adult stem cells (adipose derived stem cells, ASC). ASC have been proposed for OA treatment^2^ because of their high availability in adipose tissue, where precursor cells are nearly 300 fold more abundant than in bone marrow, which makes their recovery a simple and minimally invasive procedure. However, rather than acting as a source of precursors for regenerative purposes, there is current evidence of a prevalent anti-inflammatory, immunomodulatory and trophic activity of ASC when injected into the joint^3^. A likely scenario is that they direct regeneration starting from cartilage mesenchymal progenitors cells^4^. Indeed, after several preclinical *in vitro* and *in vivo* investigations, many clinical trials have been issued worldwide, some of them still ongoing, and listed at Clinical Trial website (https://clinicaltrials.gov/ct2/results?cond=Osteoarthritis%2C+Knee&term=adipose+derived+stem+cells&cntry=&state=&city=&dist=).

On the other hand, recently published findings have indicated that ASC have the capability to serve as precursors for articular cartilage^5^, and that state of the art technologies such as 3-D bioprinting and scaffold derivatization hold promise for the future regeneration of the joints. However, hyaline cartilage is a specialized tissue whose homeostasis is actively kept by constraints that are lost following OA onset^6^. To date, all the attempts to reconstitute hyaline cartilage either starting from chondrocytes or from mesenchymal stem cells have invariably failed, leading to production of fibrocartilage^5^. Despite the potential of differentiating into multiple cell lineages, ASC have an inherent propensity to osteoblastogenesis^7^ while chondrogenic differentiation requires extended passaging^8^.

During skeleton development, the first step is represented by condensation of mesenchymal stem cells, but then different phenomena occur downstream so that two different types of ossification intervene in different anatomical places. In “intramembranous ossification (the process responsible for the morphogenesis of the skull bones and the lateral halves of the clavicles)” the bone is deposed directly in the mesenchymal anlagen by the newly differentiated osteoblasts. In “endochondral ossification (the process responsible for the morphogenesis of the long bones)” the bone is deposed on a cartilage template originating from the condensed mesenchyme through a process termed chondrogenesis. As reviewed elsewhere^9^, progression into one or the other pathway is driven by finely tuning the activity of different master transcription factors that may have inducing or inhibiting effects and polyamines and their metabolic enzymes are implicated in this process^9^.

Polyamines are ubiquitous and endogenous small polycationic molecules that intervene in many cellular processes, regulating cell fate and differentiation through pleiotropic effects on transcription, translation and epigenetic tuning of signaling pathways^9^. The polyamines *spermidine* (SPD) and *spermine* (SPM) derive from ornithine via the diamine *putrescine* (PUT) and from S-adenosylmethionine *via decarboxylated S-adenosylmethionine* (dcSAM); however SPM can also be interconverted to SPD and SPD to PUT so that the whole polyamine metabolic network is very dynamic. The modulation of polyamine synthesis (through ornithine decarboxylase and S-adenosylmethionine decarboxylase), is a pivotal factor in affecting the intracellular levels of these molecules and plays a role in stemness control and differentiation^9^.

A change in the levels of cellular polyamines along skeletal maturation is presumed on the basis of modulation of key metabolic enzymes, but no study has previously evaluated the content of the different polyamines in mesenchymal progenitors, chondrocytes and osteoblasts. Therefore, the first purpose of our work has been to compare the differential polyamine content in mature and immature skeletal cells. We also aimed at investigating the level of oxidative stress by assessing the basal amount of γH2AX in 3-D cultures of primary ASC, and evaluating whether it increases as a function of the age and of the body mass index (BMI) of the donors. γH2AX, the phosphorylated form of the histone H2AX, is an early marker of double strand breaks (DSBs) that occurs after exposure to genotoxic stress (UV, γ irradiation or oxidative stress) in order to organize the DNA damage response (DDR) and repair^10^ and to preserve genomic integrity. DSBs are the more dangerous type of DNA damage that can threaten genome stability and therefore cells have developed the DDR^11,12^ to obtain rapid and efficient error correction.

Finally, we investigated whether the γH2AX signal in ASC cultures could be reduced by SPD and SPM, supplemented in a concentration range that spans that of these polyamines in platelet rich plasma (PRP), a blood product often used to improve bone regeneration^13^.

The findings of the study suggest that: 1. the higher polyamine content supports the stemness of ASC; 2. these cells cultured in 3-D maintain the oxidative imprinting they experienced *in vivo*, since the amount of γH2AX increases as a function of BMI and age; 3. exogenous delivery of either SPD or SPM decreases the oxidative damage; 4. finally, the protective effect of polyamine supplementation may be related to the involvement of autophagy.

## Materials and Methods

### Human adipose tissue collection and establishment of ASC primary cultures. Collection of primary chondrocytes and osteoblasts

Samples of ASC used in the study were obtained from the same cohort of patients detailed in our previous work^7^. Adipose tissue was obtained from the subcutaneous fat accessible from the patients during hip arthroplasty. The study was conducted in accordance with the 1975 Declaration of Helsinki; informed consent was obtained from all patients before surgery and the protocol was approved by the Ethics Committee of Istituto Ortopedico Rizzoli (ASC-BONE, Prot.gen.n.ro 0009883, 31 march 2011). For each primary culture, essential information about the sex, age and BMI of the patients were recorded^7^.

In the whole study, samples were harvested consecutively in a 2–year period, without any prior selection, except obvious exclusion criteria such as the presence of rheumatic diseases, chronic infective diseases, cancer, diabetes and hemocoagulation diseases. Since this paper extends the findings presented in^7^, the samples used in the present study correspond to those that were collected at the end of that study. ASC were obtained from the Stromal Vascular Fraction with conventional procedures essentially following^14^: SVF cells were initially plated at about 150,000 and then at 8,000 cells per square centimeter for expansion. After this step cells were collected and underwent a flow cytometric analysis to evaluate the expression of CD 31, 34, 45, 271, 44, 73, 90, and 105, as detailed in^7^ to ensure that the primary ASC cultures had the correct phenotype: high CD44, CD73 and CD90 staining, negative staining for CD31, CD45 and CD271 and CD34 limited to <30% of the cells.

After expansion, the cells were used to establish micromass cultures as described below; samples from three patients were stored frozen for subsequent HPLC analysis of polyamine content.

Primary chondrocyte cultures (n = 6) were obtained from cartilage of patients undergoing knee arthroplasty essentially as described in^15^. The protocol was approved by the Ethics Committee of Istituto Ortopedico Rizzoli (OA-TARGETS, Prot.gen.n.ro 0009882, 31 march 2011).

Primary osteoblast cultures (n = 4) were established as described in^16^, from tibial plateau of patients undergoing knee arthroplasty following IOR Committee approval.

### HPLC analysis of intracellular polyamine levels

HPLC analysis was carried out as described in^7^ in acidic cellular extracts after derivatization with dansyl chloride^17^. Values were expressed as nanomoles per million cells.

### Establishment of 3-D cultures of ASC

As previously detailed, the osteogenic inducing effect of polyamine addition on ASC cultured in 3-D was at first tested using different media^7^. 3-D culture of mesenchymal stem cells is a widespread method to investigate the differentiation properties of cells of different sources^18,19^. Since previous findings showed that polyamine addition promoted induction and functional activation of key transcription factors notwithstanding the type of medium used, we chose the standard medium (Dulbecco’s minimal essential medium (D-MEM) high glucose, 10% fetal calf serum (FCS) and 50 µg/ml ascorbic acid) since it allowed to appreciate a more marked change following polyamine addition.

In the present work we aimed at evaluating the effects of a range of concentration of the two main polyamines^7^, essentially corresponding to the amount found in platelet rich plasma, a blood derivative known to increase bone regeneration^13^. Therefore, 5, 7.5 or 10 µM of either spermine or spermidine were added at the time of micromass seeding in the presence of 1 mM aminoguanidine to control any toxicity of the polyamines due to their oxidation by the amino oxidase present in the bovine serum. Briefly, 250,000 cells per tube were centrifuged (740 g, 10 min at 4 °C) in 500 µl of medium so that they formed a flattened pellet which was left to mature and release ECM proteins over 1 week with medium change every second day. Common additives were represented by 100 U/mL penicillin and 100 µg/mL streptomycin. Triplicate micromasses were established for each condition.

### Western blotting of γH2AX content and protective factors in the micromasses

At 1 week maturation, micromasses underwent processing for western blot analysis, essentially as described in^7^.

To achieve effective extraction of proteins, including those bound to DNA such as γH2AX, radioimmunoprecipitation (RIPA) buffer was supplemented with 100 U/mL benzonase and 1:200 protease inhibitor cocktail (PIC; Sigma-Aldrich). 20 µl were used for each micromass. Briefly, total cellular lysates were obtained by solubilizing micromasses with RIPA buffer in addition to a vigorous homogenization with disposable pestles (Sigma) and vortexing.

A volume of micromass lysates corresponding to half micromass was loaded in the wells of Nu-Page precast 4%–10% polyacrylamide gels (Invitrogen), which were subsequently transferred onto polyvinylidene fluoride membranes by a dry electroblotting method using I-Blot (Invitrogen). Lysates referring to the samples of the same patients were loaded in the same gel and blotted to the same blot, so that the effect of 5, 7.5 and 10 µM spermidine or spermine was referred to the level of γH2AX in untreated conditions (NS). Then, the blots underwent immunodetection by exploiting the SNAP-ID device (Merck Millipore). Signals were detected with appropriate secondary antibodies and revealed with ECL Select (Amersham), using the CCD camera acquisition system of an Image Station 4000 MM coupled with the Carestream Molecular Imaging Software 5.0 (Carestream Health, Inc.). The software allowed for an accurate and automatic assessment of the molecular weight of the bands, exploiting a proper molecular weight marker (Novex Sharp Pre-stained Protein Standard, Invitrogen). The following primary antibodies were used: γH2AX (phospho-histone H2AX, Ser139, mouse monoclonal, Upstate–Millipore), beclin-1 (mouse monoclonal, BD Biosciences), LC3B (rabbit polyclonal, Novus Biologicals) and SQSTM1/p62 (mouse monoclonal, Santa Cruz Biotechnology). β-actin (mouse monoclonal, Sigma) served as loading control. Appropriate anti species antibodies and HRP conjugated were from Jackson laboratories.

Semi-quantitative analysis of band intensity was performed considering “optical density” values and using QuantityOne software (BioRad)^20^. γH2AX intensity of each ASC sample in basal conditions and after treatment with a range of polyamine concentrations were normalized differently. The “basal” level of γH2AX, i.e. the signal corresponding to that of micromasses cultured in standard medium for 1 week without treatment, was estimated after normalization of the band intensity to that of the loading control (β-actin) and then expressed as a ratio (γH2AX/β-actin) compared to that of staurosporine-treated chondrocytes (500 nm staurosporine for 8 h on chondrocytes plated at 15,000 cell/cm^2^) loaded in the same blot, 100,000 cells per lane. This “calibration” sample was loaded in all the gels, one for each patient, and allowed the inter-assay normalization. The effect of polyamine addition was instead measured in a similar way, i.e. as a ratio of γH2AX to that of β-actin. This number was then expressed as Fold changes and calculated for each condition with reference to control samples put as 1.

Assessment of the level of major markers of autophagy was carried out as stated before.

### Evaluation of autophagy promoting effects of spermidine at the single cell level

Assessment of increased autophagy requires the evidence of increased autophagosome formation, and this is usually carried out with either immunoblotting^21^ or with direct determination by immunostaining^22^. Therefore, to confirm the autophagy promoting effects of the two polyamines, a parallel set of 3-D samples in the same conditions analyzed with western blot (NS, 5, 7.5, 10 µm SPM and 5, 7.5, 10 µm SPD) were embedded in OCT at the end of 1 week maturation, as described in^7^, and kept frozen until the time of processing. Serial 5 µm sections were obtained from each micromass and placed onto sylanized glass slides, 2 sections per glass slide and stored frozen, wrapped in aluminum foil until the time of immunostaining.

The immunostaining experiment was set up using one glass slide for each condition (two micromass sections). The tissue sections were fixed with 4%PFA for 20 min, and post fixed with 90% methanol on ice for 10 min. Fixation with methanol is required to get rid of most of the LC3 proteins that are not bound to membranes, and therefore are not inserted in autophagosomes. Then a step of additional permeabilization and antigen retrieval was carried out with 20 min treatment with 0.02 U Chondroitinase ABC in 50 mM Tris/HCl, pH 8. After a brief rinsing (5 min in TBS at RT) the sections underwent blocking of non specific bindings (5% BSA (bovine serum albumin), 5% normal donkey serum and 0.1% Triton in TBS for 30 min at RT) and then washed again. Then, LC3B-II staining was performed with 5 µg/ml rabbit anti-LC3B antibody (NOVUS BIOLOGICALS # NB100-2220) along with β-Actin staining (5 µg/ml mouse monoclonal, Sigma #A2228). The antibodies were diluted in TBS + 3% BSA + 0.1% Triton and kept overnight at 4 °C. After extensive rinsing in TBS (4 × 15 min) the signals were visualized with secondary antibodies diluted in TBS + 3% BSA + 0.1% Triton: 15 µg/ml Alexa Fluor® 555 donkey anti-rabbit and 15 µg/ml Alexa Fluor® 647 donkey anti-mouse IgG secondary antibody conjugates (Novex), incubated for 30 min at RT together with 1:1000 SYTOX Green Dead Cell Stain (INVITROGEN) for nuclear counterstaining. This step was followed by extensive washings in TBS (4 × 15 min). Finally, the samples were mounted with the addition of an anti-fading reagent (1% 1,4 Diazabicyclo (2.2.2) Octane (DABCO, SIGMA) in 90% glycerol in 0.1 M pH 8.0 Tris-HCl), sealed with nail polish and stored refrigerated and in the dark for subsequent analysis at the confocal microscope.

The acquisition of Alexa 555-labelled anti-LC3B, Alexa 647-labelled anti-β-actin and Sytox Green-labelled DNA signals was performed using an A1-R confocal laser scanning microscope (Nikon), equipped with a Nikon 60×, 1.4 NA objective, and with 489, 563 and 646 nm laser lines to excite Sytox Green (green), and Alexa 555 (red) and Alexa 647 (far red) fluorescence signals, respectively. Images were taken at 2x zoom.

Emission signals were detected by a photomultiplier tube (DU4) preceded by emission filters BP 525/50 nm and BP 595/50 nm and BP 700/75 nm for Sytox Green, Alexa Fluor 555 and Alexa 647, respectively. Laser scanning, image acquisition and processing were performed with Nikon Imaging Software NIS Elements AR-4 (Nikon Inc., USA)^23^. Optical sections were spaced *0.5 µm along the z axis and were digitized with a scanning mode format of 1024 × 1024 pixels (each pixel corresponding to 100 nm, which is below the resolution limits in both the the xy plane (resolution limit: 200 nm) and in the z axis (resolution limit: 500 nm)), 4096 gray levels. Both optical sections and magnified 3-D projection images were obtained.

Signals were originally acquired as black and white and visualized with pseudocolors: nuclear counterstaining > blue (Sytox Green); LC3B-II > green (Alexa Fluor 555); β-actin > red (Alexa Fluor 647). For consistency among the different conditions, images were taken in the peripheral area of the sections.

### Assessment of activity of effector caspases

The extent of activation of the effector caspases (caspases 3/7 and caspase 6) was evaluated by mean of caspase-Glo reagents (Promega) followed by luminescent assessment of the activity, essentially as described in^24^ using 2 µl out of the 20 µl micromass lysates prepared for western blot as detailed above. 2 μl of the extract were diluted with PBS to the volume of 75 μl, joined to the same volume of substrate and left to incubate for 30 min. Then the luminescent signal was detected with a Tecan M200 luminometer. Values were normalized to the protein content, evaluated by mean of the NanoOrange Protein Quantitation Kit (ThermoFisher), following the instructions of the manufacturer.

### Statistical analysis

To correlate the γH2AX signal with either age or BMI or their combined contribution, the above data were divided in ranks. To assess the rank for each value, the range interval of either age or BMI was divided in three and a rank of 1, 2 or 3 was assigned, according to the position of the value. To take into account the synergistic activity of age and BMI, for each patient, the product of the ranks corresponding to each of the two information was correlated with γH2AX signal.

Results were expressed as the mean ± SD of different cell samples, unless otherwise stated in each legend. Comparison of different groups of samples was performed by mean of the Student’s t-test, either for paired or unpaired samples, where appropriate. Correlation between the products of the ranks assigned to age and BMI and γH2AX signal intensity was assessed by mean of the Pearson’s r. Tests were considered significant when P < 0.05, with *P < 0.05; **P < 0.01; ***P < 0.001.

## Results

### Polyamine content is higher in ASC compared to mature skeletal cells

HPLC analysis was undertaken on pellets of frozen cells that had undergone acidic extraction. The samples were derived from multiple patients. Three different cell types were analyzed: ASC, chondrocytes and osteoblasts.

**Figure 1:**
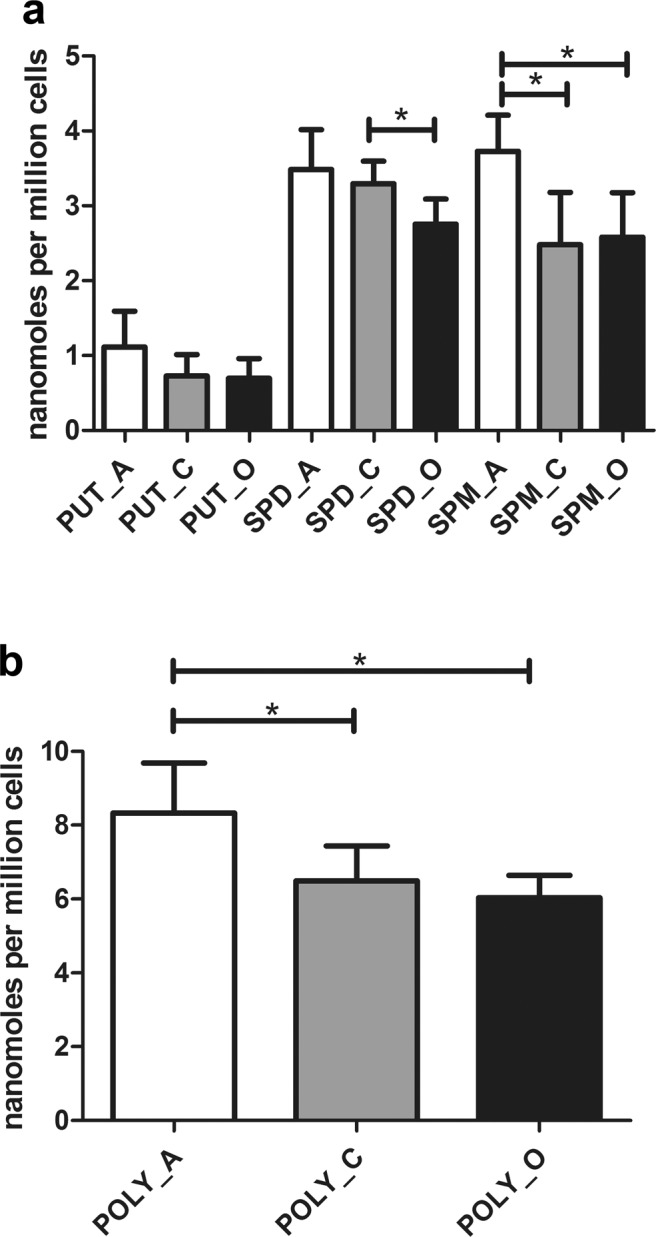
Differential polyamine content in primary cultures of adipose derived stem cells (ASC, indicated with A, 3 cultures derived from different patients) and skeletal mature cells (chondrocytes: C, 6 cultures derived from different patients and osteoblasts: O, 4 cultures derived from different patients). Analysis was performed on acidic cellular extracts after derivatization with dansyl chloride and expressed as nanomoles per million cells. (**a**) The content of each polyamine in the three different cell types and (**b**) total polyamine amount (PUT + SPD + SPM) for each cell type. Data are reported as mean ± SD. *p < 0.05, Student’s t test for unpaired data.

Results are presented in Fig. 1. ASC showed the highest content of each polyamine, in keeping with their higher differentiation potential as mesenchymal stem cells. Total polyamine content was lower in chondrocytes and in osteoblasts. Notably, SPD level in chondrocytes was significantly higher than in osteoblasts. Mean SPD/SPM ratio (±SD) was 0.94 (±0.13) for ASC, 1.42 (±0.48) for chondrocytes and 1.09 (±0.19) for osteoblasts.

### Basal level of γH2AX in 3-D culture of ASC as a function of age and BMI

Exploting immunofluorescence, we previously showed that a higher nuclear intensity of γH2AX signal is found in micromasses established from older patients^7^. However, in our particular 3-D model, western blot is more useful to estimate the total accumulation of the γH2AX foci compared to immunofluorescence that can be performed on only few sections of the 3-D construct. Therefore, in the present work we aimed to perform additional and complementary investigations to quantitatively assess γH2AX signal by mean of western blot, and to evaluate this oxidative DNA damage marker as a function of age and obesity.

**Figure 2:**
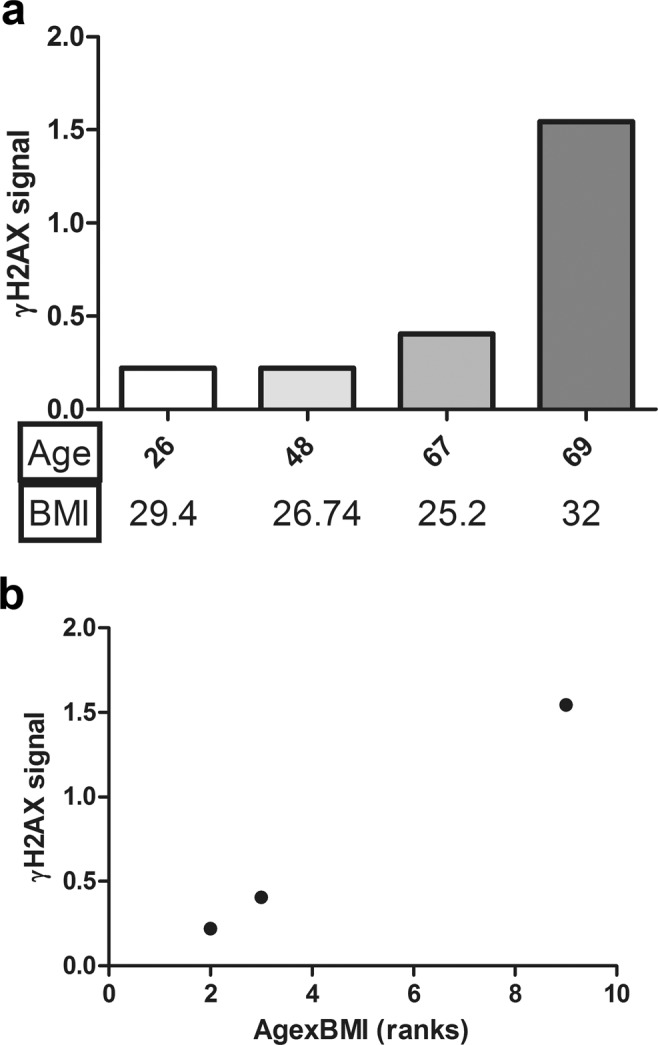
Differential levels of γH2AX detected in 1 week micromasses established from ASC from four different patients. Quantification was performed by western blot as described in Methods. (**a**) γH2AX signal was represented as a function of age. Mention of the BMI is reported below. (**b**) γH2AX signal for each patient reported as a function of the product of the ranks of both age and BMI. Two patients are overlapped, since they have the same level of γH2AX signal and 2 as the product of age and BMI ranks. The correlation parameters found with Pearson’s: r = 1, p < 0.001.

In Fig. 2(a) each of the four samples analyzed is represented together with details of age and BMI. It is noteworthy that samples derived from patients with essentially the same age (67 and 69 years) have a markedly different level of γH2AX signal according to their belonging to normal weight or class I obesity. Therefore, we investigated whether a correlation exists among the γH2AX signal and either age or BMI of the four patients analyzed. Because of the small size of our patient sample, correlation analysis of γH2AX signal with either age or BMI failed to reach a statistical significance, although in both cases we found a weak correlation, yet stronger with BMI (γH2AX signal and age: r = 0.64; γH2AX signal and BMI: r = 0.75). To assess the combined contribution of both age and BMI, synergistic factors that determine systemic oxidative damage and cell aging^6^, we categorized in three ranks the data of age and BMI, and took into account the products of these ranks, as described in Table 1.

**Table 1:** Data of BMI and age of the patients, and rank^a^ assigned.

Patients	Age(rank)	BMI (rank)	Age x BMI (ranks)
1	26 (1)	29.40 (2)	2
2	48 (2)	26.74 (1)	2
3	67 (3)	25.20 (1)	3
4	69 (3)	32.00 (3)	9

^a^Description of how the ranks were assigned is in Materials and Methods.

Indeed, γH2AX signal was found to perfectly correlate with these values (Pearson’s r = 1, p < 0.001) as shown in Fig. 2(b). Furthermore, two samples derived from patients with the same age x BMI product had the same γH2AX signal.

### Exogenous delivery of polyamines reduces γH2AX level

**Figure 3:**
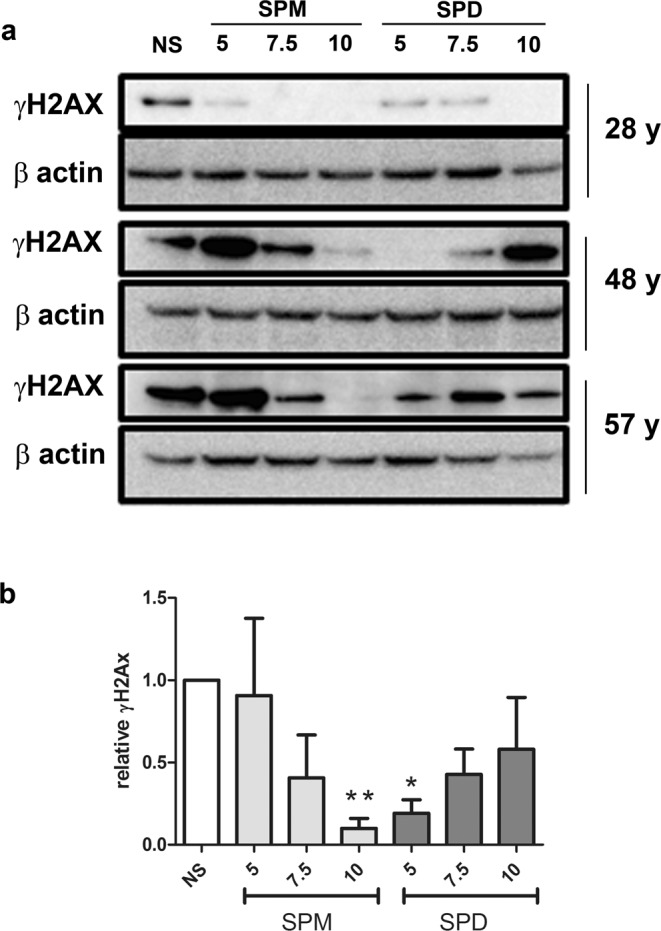
Differential effects of a range of polyamine concentration on γH2AX signal in 1 week micromasses established from ASC obtained from 3 different patients (age: 28-, 48- and 57-years old). ASC cultured in micromasses were kept for one week either in the absence (non-stimulated, NS) or in the presence of either spermine or spermidine (5, 7.5 or 10 µM). (**a**) At the end of the week of culture, micromass samples derived from single patients (of age indicated as “y”) underwent western blot analysis to stain γH2AX signal and β-actin as a loading control. The figure reports crops of these signals as detailed in Supplementary Fig. 1 that also shows the corresponding full length blots. (**b**) The signal was detected, quantified, normalized and compared versus the unstimulated conditions as described in Materials and Methods. Data are reported as mean ± SEM. *p < 0.05, **p < 0.01.

To evaluate the effects of polyamine delivery on the oxidative damage of ASC cultured in 3-D, cells derived from three different patients were used. Micromasses were established in standard medium and cultured either with or without the addition of a polyamine concentration included in a range covering that found in PRP^7^. Spermine addition consistently led to a progressively decreasing γH2AX signal that was almost undetectable at 10 µM concentration (Fig. 3a). Spermidine was also effective, but with an opposite pattern leading to a strong reduction of the γH2AX signal at the lowest concentration tested. Figure 3b reports the cumulative data obtained for the three patients analyzed. The data obtained from samples treated with polyamines were compared to those of unstimulated conditions by statistical analysis: a significant difference was found for 10 µM spermine and 5 µM spermidine with respect to untreated controls.

### Exogenous delivery of polyamines impacts on markers of autophagy

**Figure 4:**
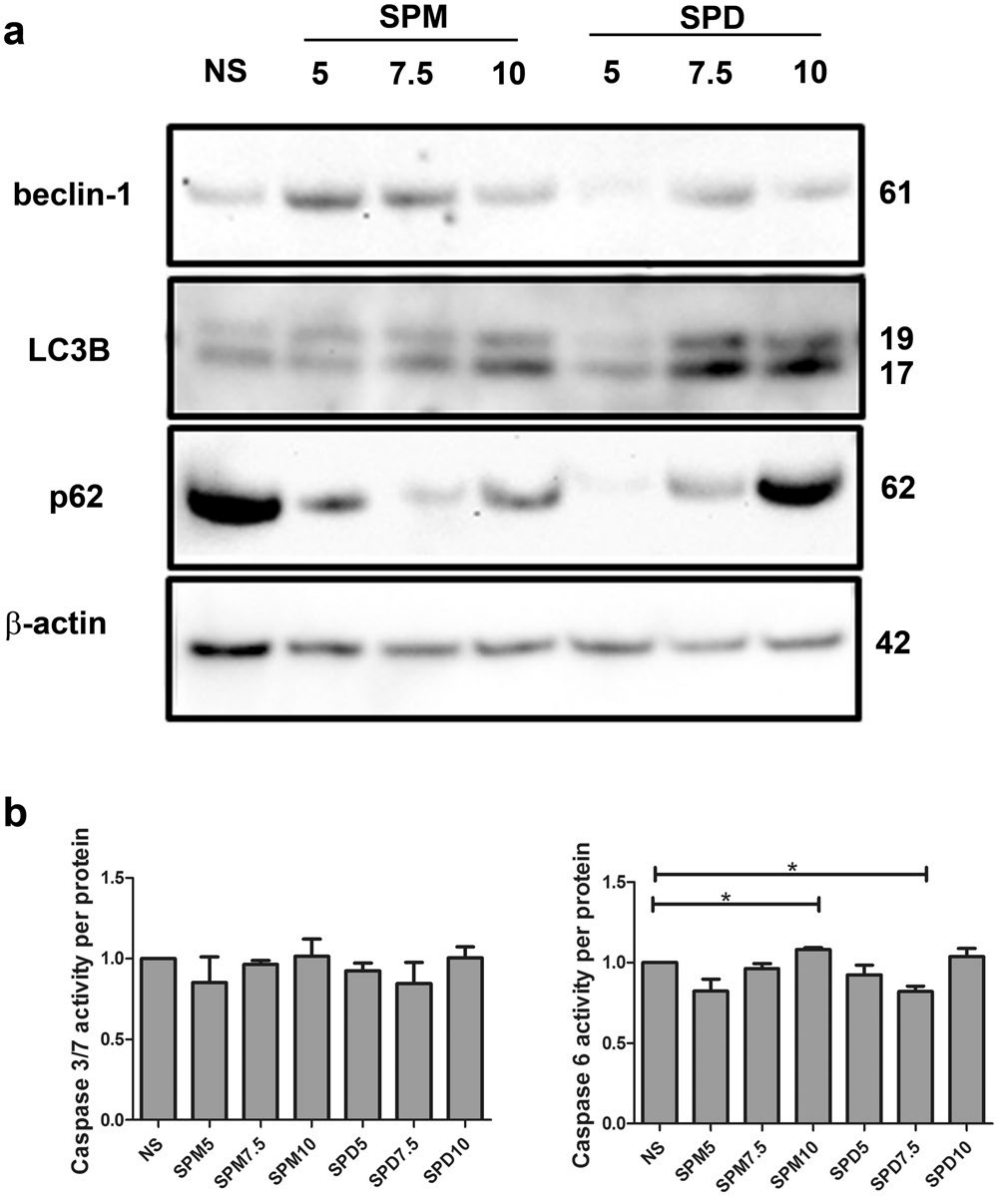
(**a**) Differential effects of a range of polyamine concentration on autophagic proteins in 1 week micromasses established from ASC. ASC were cultured in micromasses for one week either in unstimulated conditions (NS) or in the presence of either spermine or spermidine (5, 7.5 or 10 µM). At the end of the week of culture, the samples underwent western blot analysis to stain beclin-1, LC3B and SQSTM1/p62. β-actin served as a loading control. The figure reports crops of these signals as detailed in Supplementary Fig. 2 that also shows the corresponding full length blots. Molecular weight of each protein is indicated on the right side of the image. One representative case out of three analyzed is shown. (**b**) Effects of polyamines on the activity of effector caspases 3/7 and 6 in micromasses from three different patients. Data are reported as mean ± SEM. *p < 0.05.

In order to explore possible intracellular pathways involved in the effects of polyamines on the levels of γH2AX, we took into consideration some markers suggestive of cell protective activities, and in particular the markers of autophagy. The level of different proteins involved in this process, i.e. beclin-1, LC3 and p62, was evaluated by western blotting. Results relative to one representative patient are shown in Fig. 4a.

The first step in autophagy and in autophagosome formation is the onset of the “isolation membrane” a double membrane structure able to enwrap misfolded proteins or damaged organelles. Beclin-1 acts as a molecular platform to regulate this initiation event, and therefore has a pivotal role in promoting the autophagic process and in contrasting apoptosis^25,26^. We found that SPM and SPD treatment generally increased beclin-1 level with the exception of samples corresponding to 5 µM SPD treatment. It is noteworthy that the autophagic flux may impact on the level of some autophagic proteins, including beclin-1, since they are involved particularly in mitophagy, as previously reported^27^.

Autophagosome elongation and maturation require LC3 intervention. The key regulatory step of autophagy is autophagosome formation, allowed by LC3 post-translational modification, i.e. the accumulation of a membrane-associated, phosphatidylethanolamine (PE)-conjugated form of LC3 (LC3-II), easily distinguishable from the parental protein by western blot^21^. The use of anti-LC3B antibodies allowed us to stain the type of autophagosome found more abundant throughout the cytoplasm and less at the nuclear level, and therefore more likely corresponding to those in charge of disposing off damaged organelles and molecules, according to recent literature^28^. The number of autophagosome**s**, and therefore the extent of autophagy, is correlated to the amount of LC3-II, i.e. the LC3-I protein after PE-conjugation, whose level is however also dependent on the efficiency of the autophagic flux. Guidelines for the correct evaluation of LC3 immunoblotting have been carefully detailed by^21^ who suggested some methods for the assessment of the autophagic flux. Given the nature of our samples (3-D cultures kept for 1 week in different culture conditions) the long-term use of lysosomal protease inhibitors was impractical and therefore we chose to assess the the autophagic flux by evaluating the level of the SQSTM1/p62 protein. SQSTM1/p62, an autophagic cargo adapter, has the ability to bind to the PE conjugated form of LC3 to allow immobilization of various ubiquitinated proteins and organelles into the autophagosomes, and undergoes itself degradation when the flux is not inhibited. Therefore its reduction points at the effective completion of the autophagic flux. Evaluation of the opposite variations in LC3B-II and p62 amounts indicated that SPM and SPD exogenous administration stimulates the autophagic flux, as also in keeping with a relative higher intensity of LC3B-II over LC3B-I.

### Exogenous delivery of polyamines has only limited effects on caspase activity

Effector caspase activities were also evaluated in polyamine supplemented micromasses. The results presented in Fig. 4b show no significant effect for major effector caspases 3/7, ruling out the involvement of apoptosis in polyamine protective actions. A statistically significant increase was found for caspase 6 in 10 µM SPM-treated samples, and a significant decrease in 7.5 µM SPD-treated samples, but these differences were rather small.

### Immunostaining confirms that polyamine addition increases autophagy

**Figure 5:**
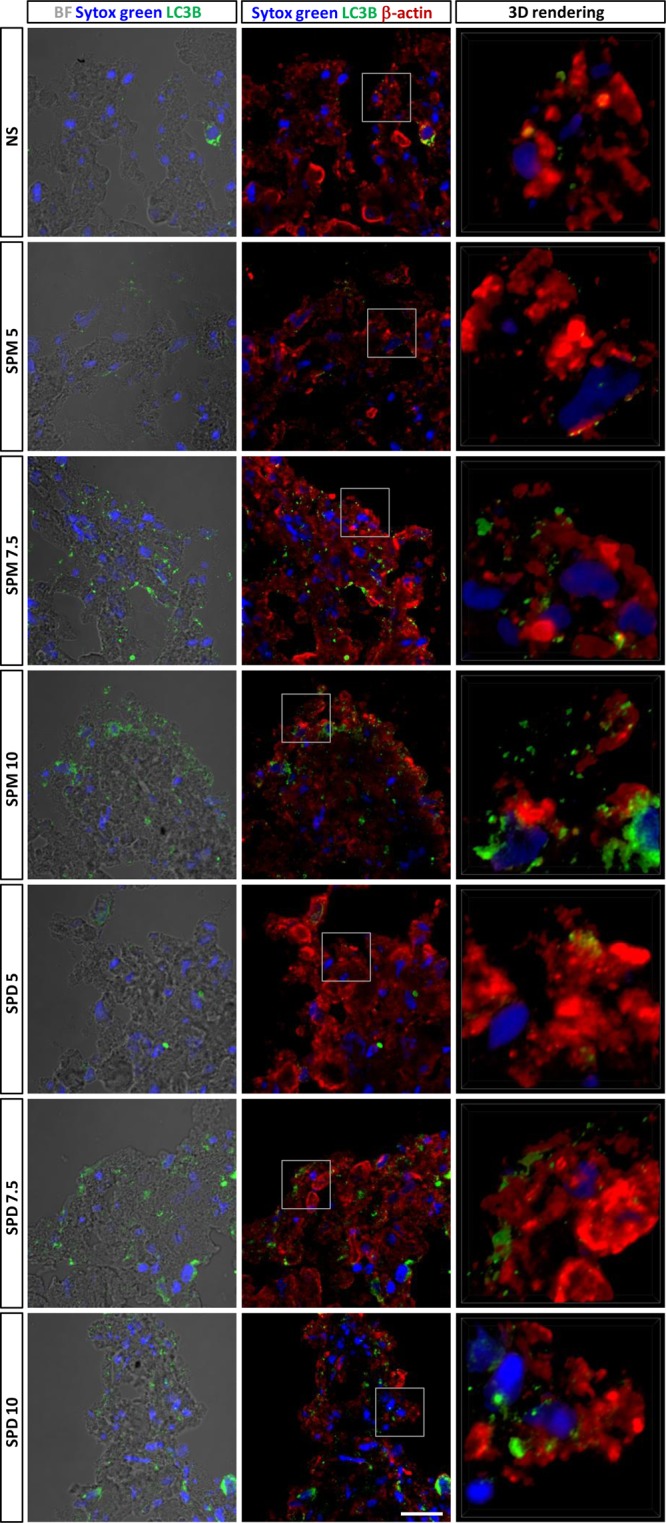
Confocal analysis of the accumulation of autophagosomes in micromasses grown with a range of polyamine concentration. Signals were originally acquired at high sensitivity as gray levels and then visualized with pseudocolors: nuclear counterstaining > blue (Sytox Green); LC3B-II > green (Alexa Fluor 555); β-actin > red (Alexa Fluor 647). Addition of polyamine increases the level of autophagy in the 3-D cultures as assessed by mean of increased accumulation of LC3B-II positive dots. Left pictures: bright field images informative of the tissue architecture, with superimposition of the nuclear counterstaining and of the LC3B-II signal; middle pictures: confocal images with nuclear counterstaining, LC3B-II and β-actin signals and finally, right pictures: high magnification 3-D rendering of small areas of the previous images (as indicated by the square frame). Bar = 200 μm.

Immunostaining experiments confirmed that the addition of polyamine increases the level of autophagy in the 3-D cultures as assessed by mean of increased accumulation of LC3B-II positive dots i.e. autophagosomes (Fig. 5). Furthermore, the amount of these signals increased with the polyamine concentration, thus matching the pattern of the 17 kDa LC3B-II bands in western blot. For each sample, three different images were collected: bright field images informative of the tissue architecture, with superimposition of the nuclear counterstaining and LC3B-II signal; confocal images with nuclear counterstaining, LC3B-II and β-actin signals and finally, high magnification 3-D rendering of small areas of the previous images.

## Discussion

This study reports novel findings concerning polyamine biology in ASC with reference to their use in musculoskeletal engineering. Firstly, we measured the intracellular content of polyamines and found that it is higher in these mesenchymal stem cells compared to mature skeletal cells such as chondrocytes and osteoblasts. These differences about polyamine levels could be connected to the stemness of ASC and their differentiation toward more mature phenotypes, since polyamine delivery is known to decrease dcSAM and consequently increase methyltrasferase activity^29^. Indeed, it has been shown that the pattern of DNA hypomethylation may represent a signature that allows to distinguish specific tissue types and that may be recapitulated during *in vitro* directed differentiation^30^. In keeping with this information, the three cell types examined in our study (mesenchymal stem cells, chondrocytes and osteoblasts) have progressively decreasing content of polyamines. In particular, osteoblasts have a lower content of spermidine compared to chondrocytes. Indeed, we previously showed that spermidine addition to 3-D cultures of chondrocytes is able to induce SOX-9 expression, the master transcription factor that supports their phenotype^31^. Therefore, it could be conceivable that the statistically significant higher spermidine content in chondrocytes compared to osteoblasts could at the same time support the maintenance of the chondrocyte phenotype, while its lower content in osteoblasts could support the progression towards the more mature cells in the chondrogenesis > endochondral ossification axis, thanks to a DNA demethylating effect. It has indeed been recognized that in endochondral ossification, besides bone formation due to recruitment of mesenchymal stem cells undergoing osteoblastogenesis, in the final stages some osteoblasts originate from chondrocytes by mean of a transdifferentiation process^32^. Interestingly, it has been reported that delivery of polyamines (either *in vivo* by mean of a high polyamine containing diet or *in vitro* by medium supplementation) has the ability of recovering the normal methylated status of the DNA, that is a requirement for human health and longevity^29^. This is in keeping with epidemiological studies that indicate that the healthiest dietary regimens (the Mediterranean and the Japanese diets) feature a high polyamine content^33,34^.

We already approached the biology of ASC using 3-D cultures^7^ that more closely mimic the complex environment of cells embedded in their native extracellular matrix (ECM)^35^ and that enable to obtain more meaningful information compared to most conventional studies carried out in 2-D. In our previous study we demonstrated the effects on cell differentiation of polyamine supplementation: 5 µM spermine enhanced ASC osteoblastogenesis from the early to the mature phase, along with a pro-survival effect^7^. However, in regenerative medicine strategies, beyond the effects on cell differentiation, a critical issue is represented by the maintenance of the pool of precursors and their cellular homeostasis. Therefore, in the present study we chose the 3-D culture model to evaluate the degree of DNA oxidative damage as a function of the age and of the BMI of the donors, and the effects of polyamine addition. Aging and obesity are both conditions that contribute to a systemic status of oxidative damage and low grade inflammation^6^ and represent determinants of the aging stress response^36^ that combines increased cell damage and decreased homeostatic functions. The aging stress response^36^ can be easily measured by mean of γH2AX detection^37,38^.

We found that the level of γH2AX strongly correlates with the combination of the age and of the BMI of the patients. Obesity indeed anticipates failure of homeostatic mechanisms in many post mitotic tissues, such as chondrocytes, so that a distinct phenotype of OA (metabolic syndrome associated OA or MetOA) has been recognized in recent years^39^. ASC are also affected by this systemic status, and recent findings showed that cells derived from obese patients have reduced viability and proliferative potential besides mitochondrial defects supported by metabolic changes that further boost an oxidative environment^40^. ASC derived from obese human and murine subjects have higher percentages of cells in early and late apoptosis^40^. Literature observations have shown that ASC culture itself in atmospheric O_2_ leads to accumulation of oxidative damage^41^. Moreover, we previously reported that the level of γH2AX was higher in 3-D cultures established with ASC derived from aged patients^7^. The latter is in keeping with the increased release of ROS and NOS from ASC of aged donors as well as with the decreased content of anti-oxidant enzymes^42^.

Interestingly, differences are emerging in the performance of ASC isolated from either subcutaneous or visceral fat and from different anatomical sites^2^ or as a function of the metabolic status of the subject, with particular reference to obesity. White adipose tissue deregulation is pivotal in metabolic syndrome. In virtue of the M2 to M1 transition of the macrophages resident in the “niche” and the overall inflammatory environment, ASC in adipose tissue of obese subjects rather than exerting anti-inflammatory activities release inflammatory cytokines, chemokines and adipokines^43^ and circulating microRNAs^44^ able to target proteins in charge of protecting the cells in conditions of metabolic/oxidative stress^45^, such as AMPKα and PPARγ. This results in increased DNA damage and senescence in ASC, thus short-circuiting their stemness^45^.

The findings of the present study, indicate that the level of DNA damage is attenuated by delivery of both polyamines in the low micromolecular range. SPD and SPM can regulate transcription by acting directly on DNA conformation and chromatin condensation. The protective role of polyamine from DNA damage is in keeping with previous findings showing that genetically induced polyamine deficiency leads to specific alterations of the cell-cycle progression and DNA damage^46^. The antioxidant activity is higher for spermine that is intimately associated with DNA^47,48^. However, more recent reports have highlighted a peculiar ability of spermidine to promote homeostatic activities such as autophagy, thus reducing cell stress and promoting longevity. In PC12 and cortical neuron cells it has been shown that spermidine addition reduces caspase activation and downstream cleavage of beclin-1, thereby restoring autophagy^26^. Spermidine sustains mitochondrial function and exerts anti-inflammatory activities thus preventing stem cell senescence^49^. This is in keeping with the recently reported ability of this molecule to promote cardioprotection in human and mice, supported by enhanced autophagy, mitophagy and mitochondrial respiration^50^.

Another recent report also evaluated the effects exerted by a range of polyamine concentration on *in vitro* gene expression, and found the latter to be enhanced at low and inhibited at high concentration, when the high level of charged polyamines leads to compaction of DNA that is no more available for RNA transcription^51^. Spermine compacts DNA more actively than spermidine^52^. Furthermore, inside the cells spermidine is primarily complexed to anionic molecules and macromolecules (phospholipids of the cell membrane, ATP, RNA, DNA wrapped with histones) and only a fraction of the intracellular content represents the “free pool”^53^. Slight variation of this pool is achieved by treatment of the cells with low- µmolar/sub-µmolar amounts of spermidine, that have been shown to promote anti-inflammatory effects via triggering of T-cell protein tyrosine phosphatase (TCPTP)^54^ or autophagy^55^. TCPTP is a phosphatase that exerts an anti-inflammatory role being able to down-regulate multiple signaling transduction pathways in the cells, acting at multiple levels^53^. An *in vitro* high throughput assay identified spermidine (at the same concentration used in the present work) and other 5 small molecules among 64280 potential candidates screened to tease out the best selective TCPTP agonists^56^. Therefore, spermidine supplementation in the low-µmolar range may at the same time rescue fundamental homeostatic activities in the cells and exert anti-inflammatory effects. Autophagy in particular has been recently recognized as pivotal in maintaining the functionality of old and young stem cells^57^, reverting a senescence status and rescuing their regenerative potential^58^. In this perspective a critical role for TCPTP in sustaining autophagy has been described in intestinal cells^59^. In our model, the combined evaluation of the autophagy related proteins (LC3-II/LC3-I ratio and p62) indicated an autophagy promoting effect that was indeed highest at 5 µM SPD, in keeping with the strongest γH2AX reduction. The complementary evaluation of autophagy and removal of markers of oxidative damage is useful in correctly interpreting autophagy, because accumulation of autophagy proteins may indicate impairment of the autophagic flux^60^. If the latter is instead enhanced, autophagy markers are reduced, together with removal of damaged molecules and organelles^21^.

Collectively, our experimental findings support the use of polyamine supplementation to rescue both homeostasis and differentiation potential of stem cells derived from adipose tissues, thus representing an appealing tool to improve regenerative approaches.

## Supplementary information

Supplementary Figures and Information
