# Supplementary material for: Polyamine supplementation reduces DNA damage in adipose stem cells cultured in 3-D
Source: Sci Rep. 2019 Oct 3;9:14269. doi: 10.1038/s41598-019-50543-z (PMC6776621; doi:10.1038/s41598-019-50543-z)
Supplement: Supplementary file 1 — Supplementary Figures and Information [file 41598_2019_50543_MOESM1_ESM.pdf]

Supplementary Figures and Information

Polyamine supplementation reduces DNA damage in adipose stem cells cultured in 3-D

Manuela Minguzzi, Serena Guidotti, Daniela Platano, Stefania D'Adamo, Silvia Cetrullo, Elisa Assirelli, Spartaco Santi, Erminia Mariani, Giovanni Trisolino, Giuseppe Filardo, Flavio Flamigni, Rosa Maria Borzì

S1

A

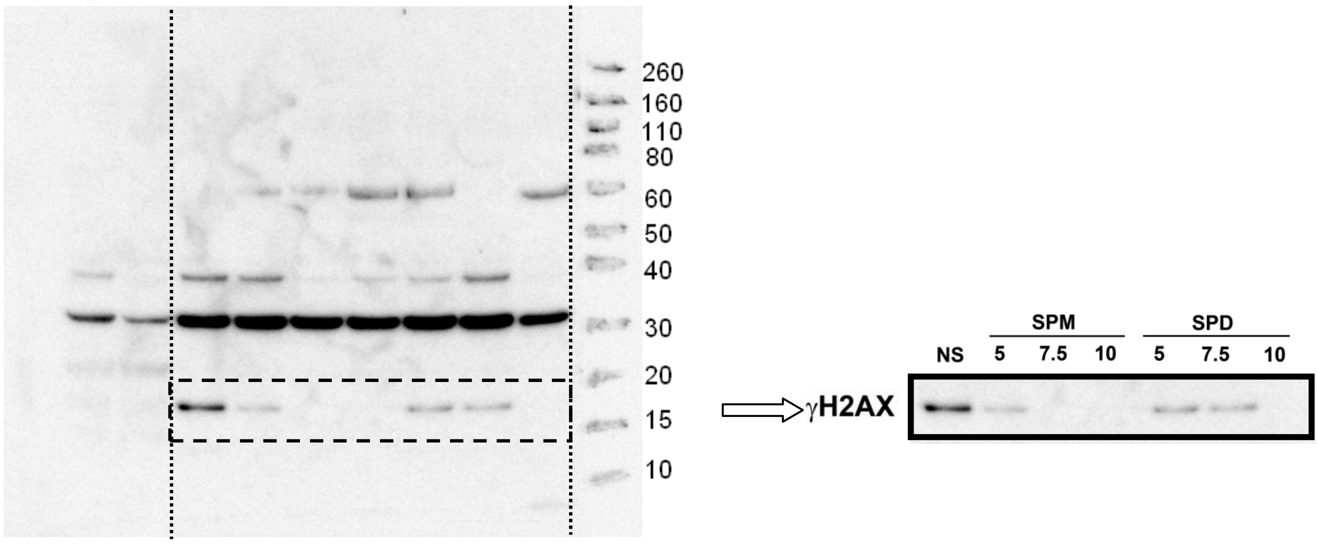
260
160
110
80
60
50
40
30
20
15
10
γH2AX
SPM
SPD
NS
5
7.5
10
5
7.5
10

B

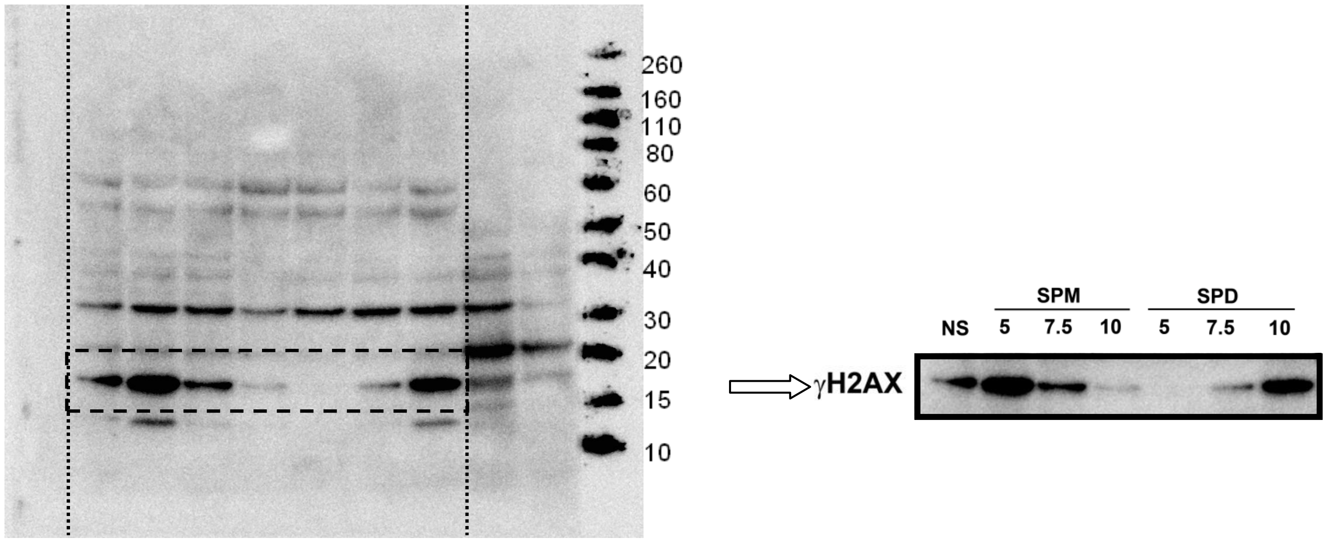
260
160
110
80
60
50
40
30
20
15
10
γH2AX
SPM
SPD
NS
5
7.5
10
5
7.5
10

C

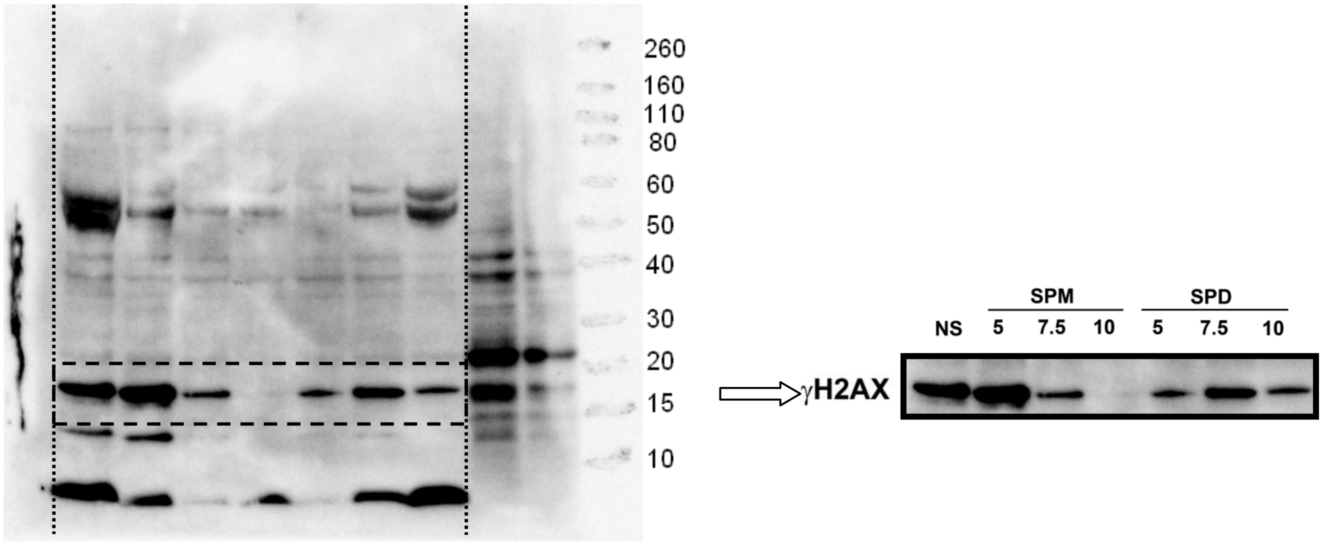
260
160
110
80
60
50
40
30
20
15
10
γH2AX
SPM
SPD
NS
5
7.5
10
5
7.5
10

D

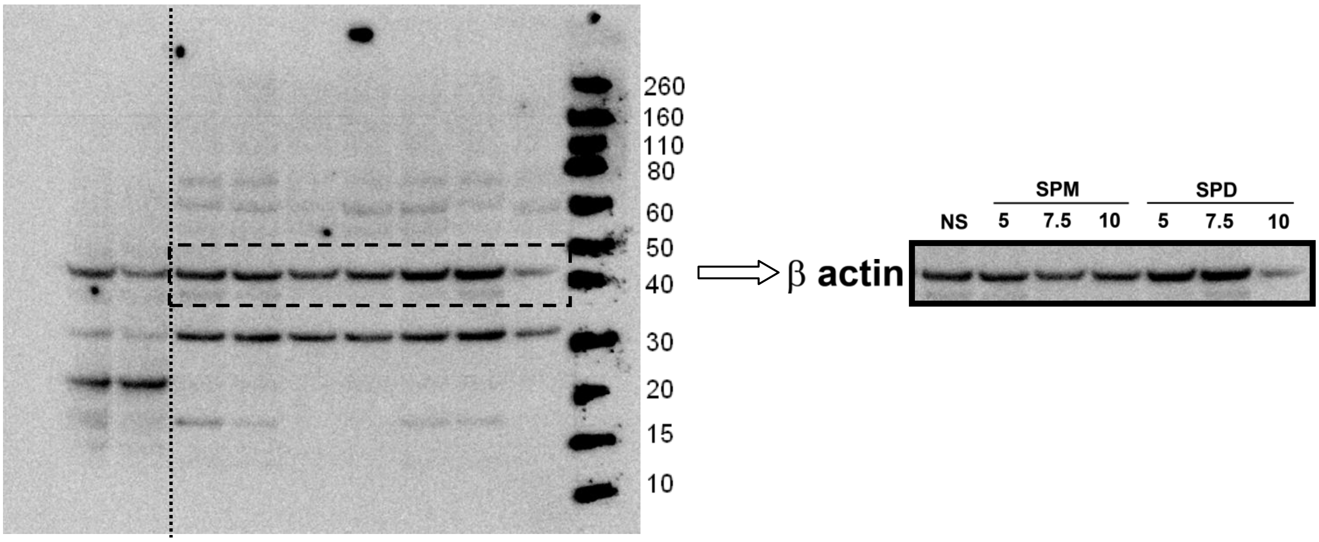
260
160
110
80
60
50
40
30
20
15
10
β actin
SPM
SPD
NS
5
7.5
10
5
7.5
10

E

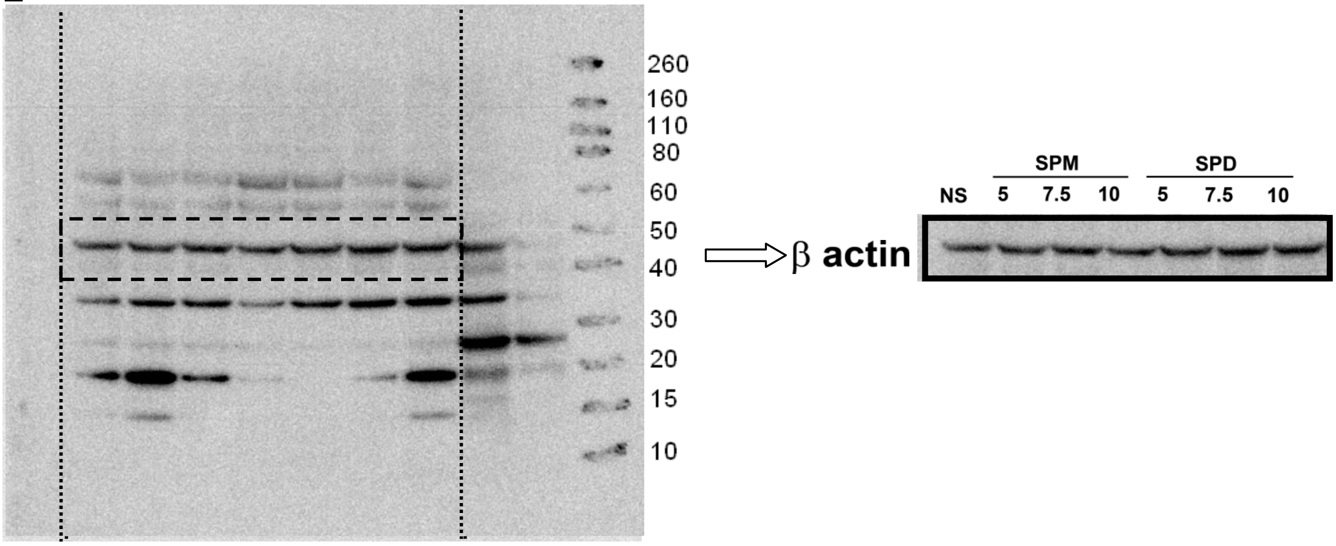
260
160
110
80
60
50
40
30
20
15
10
β actin
SPM
SPD
NS
5
7.5
10
5
7.5
10

F

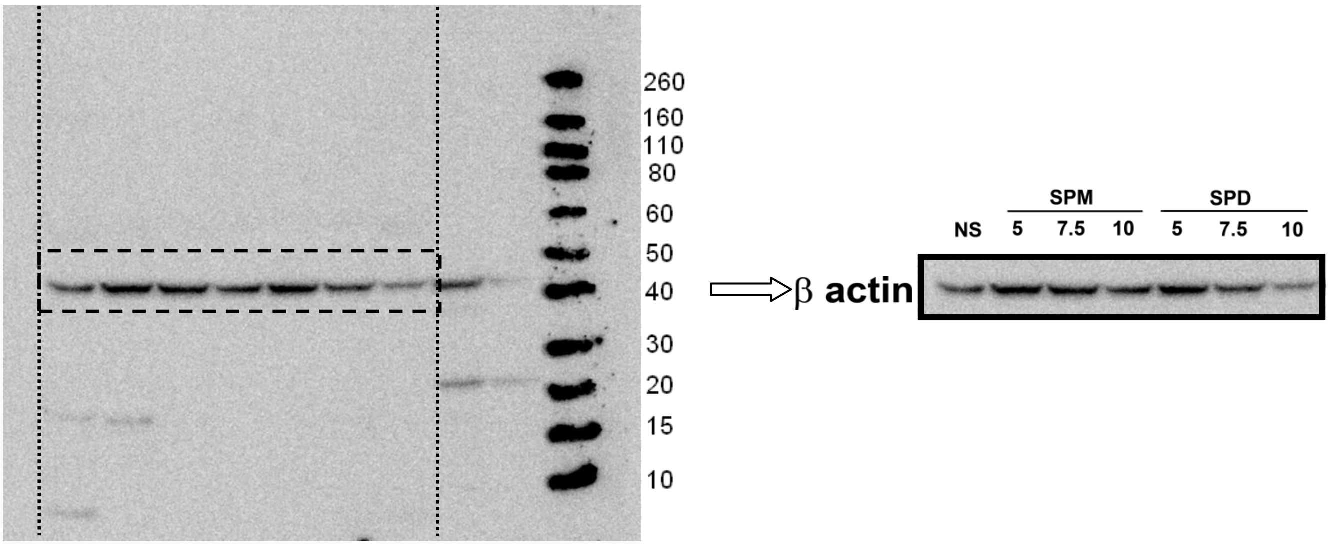
260
160
110
80
60
50
40
30
20
15
10
β actin
SPM
SPD
NS
5
7.5
10
5
7.5
10

Supplementary Figure S1.: Full blots used to derive the γH2AX results shown in Figure 3 of the main manuscript, obtained with lysates of micromasses kept for 1 week in unstimulated conditions or stimulated with either spermine (SPM) or spermidine (SPD) at 5, 7.5 and 10 µM loaded in NuPAGE Novex 4-12% Bis-Tris gels. Samples were run with NuPAGE MOPS along with Novex Sharp Pre-Stained Protein Standards. After protein transfer, the lanes containing the standards were cut from the membranes containing the samples. To assess the molecular weight of western blot stained bands the pre-stained bands of the markers were highlighted by mean of a Glow Writer pen (http://divbio.com/glow-writerpen.aspx) and at the end of western blotting, the lanes containing the Protein Standards were juxtaposed to the original membranes.
The dashed rectangles indicates the bands included in the crops, and on the right black solid rectangles indicate the bands used to set up Figure 3. In the full blots, dotted lines separate the portions of the membranes used to obtain the results presented in Figure 3.
A: Full blots used to derive the original γH2AX results shown in Figure 3 of the main manuscript, obtained with micromass lysates at 1 week established with adipose derived stem cells from a patient aged 28 years. The dashed rectangle indicates the bands included in figure 3.
B: Full blots used to derive the original γH2AX results shown in Figure 3 of the main manuscript, obtained with micromass lysates at 1 week established with adipose derived stem cells from a patient aged 48 years. The dashed rectangle indicates the bands included in figure 3.
C: Full blots used to derive the original γH2AX results shown in Figure 3 of the main manuscript, obtained with micromass lysates at 1 week established with adipose derived stem cells from a patient aged 57 years. The dashed rectangle indicates the bands included in figure 3.
D: Full blots used to derive the original β-actin results shown in Figure 3 of the main manuscript, obtained with micromass lysates at 1 week established with adipose derived stem cells from a patient aged 28 years. The dashed rectangle indicates the bands included in figure 3.
E: Full blots used to derive the original β-actin results shown in Figure 3 of the main manuscript, obtained with micromass lysates at 1 week established with adipose derived stem cells from a patient aged 48 years. The dashed rectangle indicates the bands included in figure 3.
F: Full blots used to derive the original β-actin results shown in Figure 3 of the main manuscript, obtained with micromass lysates at 1 week established with adipose derived stem cells from a patient aged 57 years. The dashed rectangle indicates the bands included in figure 3.

S2

A

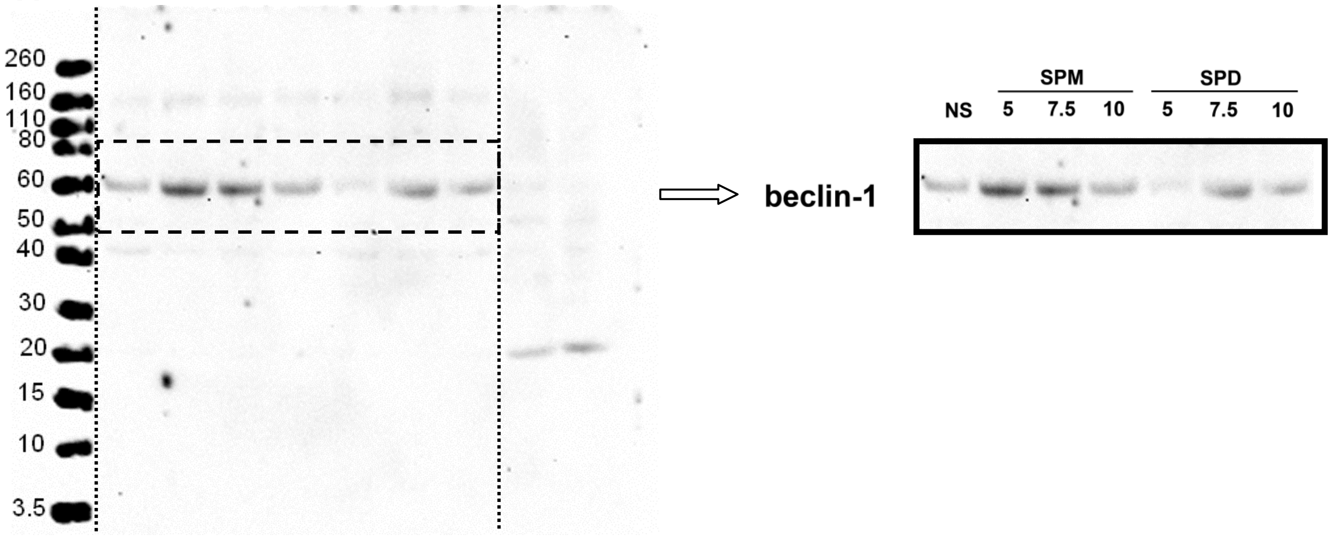
260
160
110
80
60
50
40
30
20
15
10
3.5
beclin-1
SPM
SPD
NS
5
7.5
10
5
7.5
10

B

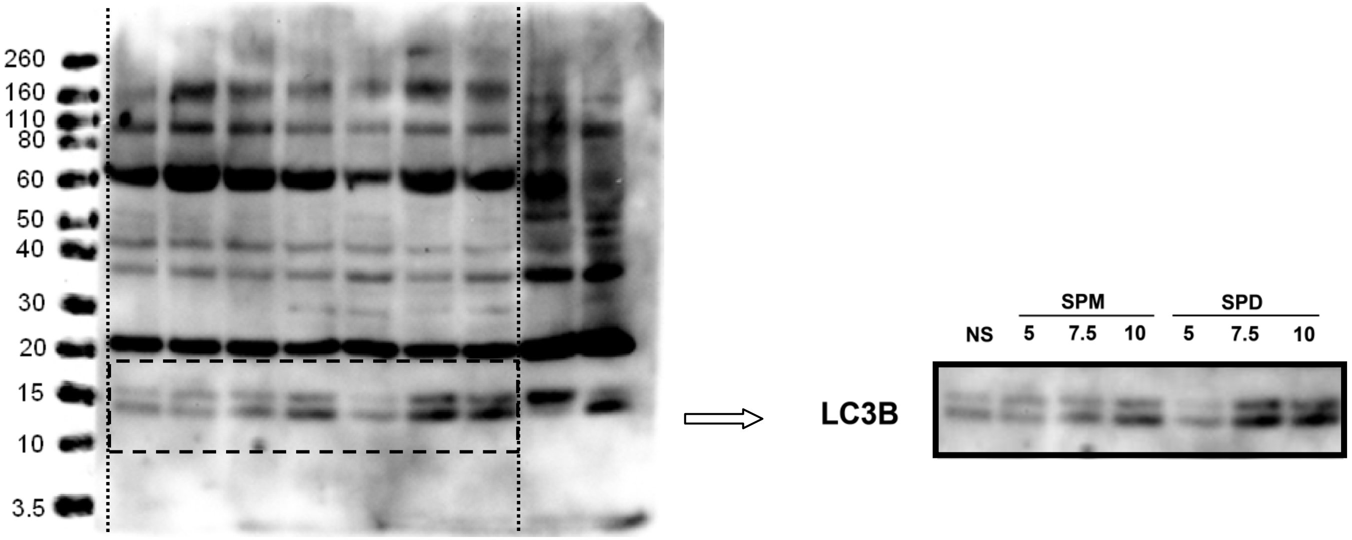
260
160
110
80
60
50
40
30
20
15
10
3.5
LC3B
SPM
SPD
NS
5
7.5
10
5
7.5
10

C

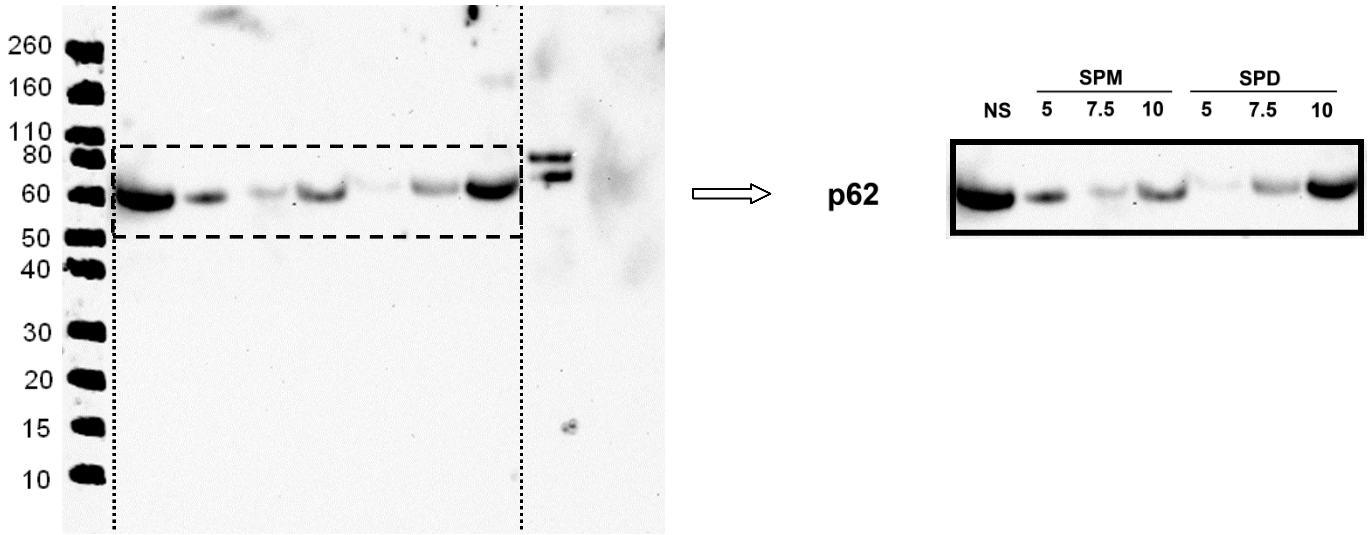
260
160
110
80
60
50
40
30
20
15
10
p62
SPM
SPD
NS
5
7.5
10
5
7.5
10

D

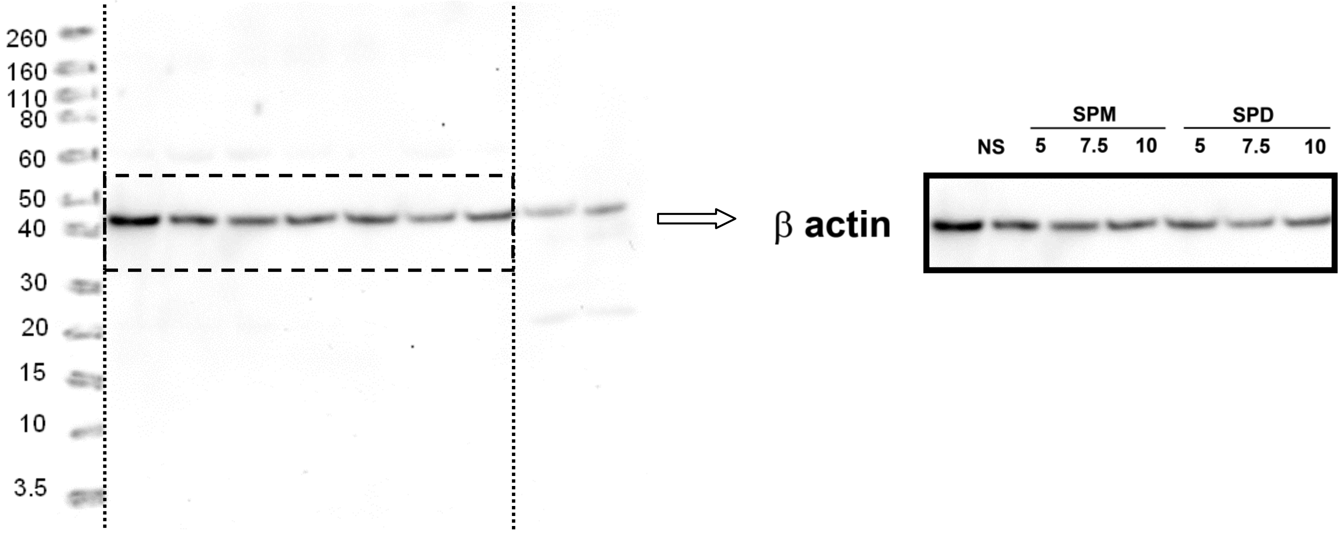

Supplementary Figure S2.: Full blots used to derive the results shown in Figure 4 of the main manuscript describing the polyamine effects on pivotal autophagic proteins. These results were obtained with lysates of micromasses obtained from one representative patient, kept for 1 week in unstimulated conditions or stimulated with either spermine (SPM) or spermidine (SPD) at 5, 7.5 and 10 µM loaded in NuPAGE Novex 4-12% Bis-Tris gels. Samples were run with NuPAGE MOPS along with Novex Sharp Pre-Stained Protein Standards. After protein transfer, the lanes containing the standards were cut from the membranes containing the samples. To assess the molecular weight of western blot stained bands the pre-stained bands of the markers were highlighted by mean of a Glow Writer pen (http://divbio.com/glow-writerpen.aspx) and at the end of western blotting, the lanes containing the Protein Standards were juxtaposed to the original membranes.

The dashed rectangles indicates the bands included in the crops, and the black solid rectangles on the right indicate the bands used to set up Figure 4. In the full blots, dotted lines separate the portions of the membranes used to obtain the results presented in Figure 4.

A: Full blot used to derive the original beclin-1 results shown in Figure 4 of the main manuscript. The dashed rectangle indicates the bands included in figure 4.

B: Full blot used to derive the original LC3B results shown in Figure 4 of the main manuscript. The dashed rectangle indicates the bands included in figure 4.

C: Full blot used to derive the original p62 results shown in Figure 4 of the main manuscript. The dashed rectangle indicates the bands included in figure 4.

D: Full blots used to derive the original β-actin results shown in Figure 4 of the main manuscript, The dashed rectangle indicates the bands included in figure 4.

	AGE	BMI	SEX	DIAGNOSIS	COUNTRY OF BIRTH AND ETHNICITY	
**1**	26	29.4	Male	Post traumatic coxarthrosis	Tunisia (caucasian)	**Used for Figure 2**
**2**	48	26.7	Female	Left coxarthrosis	Italy (caucasian)	
**3**	67	25.2	Male	Right hip post traumatic osteonecrosis	Italy (caucasian)	
**4**	69	32	Female	Right coxarthrosis	Italy (caucasian)	
**5**	28	25.7	Female	Left hip dysplasia	Albania (caucasian)	**Used for Figure 3**
**6**	48	27.3	Female	Right hip dysplasia	Italy (caucasian)	
**7**	57	28.4	Male	Right coxarthrosis	Italy (caucasian)	

Supplementary table: essential data relative to the patients who contributed the ASC used in the manuscript, distinguishing those used for Figure 2 and those used for Figure 3.
